# Next-generation sequencing identified *SPATC1L* as a possible candidate gene for both early-onset and age-related hearing loss

**DOI:** 10.1038/s41431-018-0229-9

**Published:** 2018-09-03

**Authors:** Anna Morgan, Dragana Vuckovic, Navaneethakrishnan Krishnamoorthy, Elisa Rubinato, Umberto Ambrosetti, Pierangela Castorina, Annamaria Franzè, Diego Vozzi, Martina La Bianca, Stefania Cappellani, Mariateresa Di Stazio, Paolo Gasparini, Giorgia Girotto

**Affiliations:** 1 Department of Medical Sciences, University of Trieste, Trieste, Italy; 2 Sidra Medical and Research Center, Doha, Qatar; 3 Heart Science Centre, National Heart and Lung Institute, Imperial College London, London, UK; 4 UO Audiology, Fondazione IRCCS Ca Granda, Ospedale Maggiore Policlinico, Mangiagalli e Regina Elena, Milan, Italy; 5 Audiology Unit, Department of Clinical Sciences and Community Health, University of Milan, Milan, Italy; 6 Ceinge Biotecnologie Avanzate, Naples, Italy; Istituto di Audiologia, Dipartimento di Neuroscienze, Scienze Riproduttive e Odontostomatologiche, Università di Napoli “Federico II”, Naples, Italy; 7 Medical Genetics, Institute for Maternal and Child Health - IRCCS “Burlo Garofolo”, Trieste, Italy

**Keywords:** Next-generation sequencing, Genetic association study

## Abstract

Hereditary hearing loss (HHL) and age-related hearing loss (ARHL) are two major sensory diseases affecting millions of people worldwide. Despite many efforts, additional HHL-genes and ARHL genetic risk factors still need to be identified. To fill this gap a large genomic screening based on next-generation sequencing technologies was performed. Whole exome sequencing in a 3-generation Italian HHL family and targeted re-sequencing in 464 ARHL patients were performed. We detected three variants in *SPATC1L*: a nonsense allele in an HHL family and a frameshift insertion and a missense variation in two unrelated ARHL patients. In silico molecular modelling of all variants suggested a significant impact on the structural stability of the protein itself, likely leading to deleterious effects and resulting in truncated isoforms. After demonstrating *Spatc1l* expression in mice inner ear, in vitro functional experiments were performed confirming the results of the molecular modelling studies. Finally, a candidate-gene population-based statistical study in cohorts from Caucasus and Central Asia revealed a statistically significant association of *SPATC1L* with normal hearing function at low and medium hearing frequencies. Overall, the amount of different genetic data presented here (variants with early-onset and late-onset hearing loss in addition to genetic association with normal hearing function), together with relevant functional evidence, likely suggest a role of *SPATC1L* in hearing function and loss.

## Introduction

Hereditary hearing loss (HHL) is the most common sensory disorder and can be differentially classified according to age of onset (congenital or acquired), type (conductive, sensorineural or mixed) and presence of other clinical features (syndromic or non-syndromic) [1]. It is a characterized by an unparalleled genetic heterogeneity and to date, more than 95 genes and 158 loci have been associated with non-syndromic hearing loss (NSHL), which accounts for the vast majority of HHL cases (http://hereditaryhearingloss.org/). As regards age-related hearing loss (ARHL), a multifactorial disorder and the most common sensory impairment in the elderly, an exiguous number of genes (identified using genome-wide association studies (GWAS) and animal models [2–6]) has been hypothesized as causative [7]. To date, only few studies showed the presence of genes/proteins that group into common pathways of inner ear function and loss and that are probably involved in both early-onset HHL and ARHL phenotypes (e.g., *CDH23* gene, associated with ARHL, NSHL and Usher syndrome 1D) [8]. Identifying other genetic elements causative of both early-onset and late-onset HL would be extremely valuable for a better understanding of the molecular mechanisms regulating hearing function. Herein we describe *SPATC1L*, encoding speriolin-like, a protein of unknown function, as likely involved in early-onset HHL, ARHL and normal hearing function.

## Materials and methods

Prior to study onset, approval was obtained from the Institutional Review Board of IRCCS Burlo Garofolo, Trieste, Italy. Consent forms for clinical studies were signed by each participant and all research was conducted according to the ethical standards as defined by the Helsinki Declaration. Genomic DNA for sequencing and genotyping was extracted from peripheral blood or saliva using QIAsymphony instrument (Qiagen) and the Oragene kit (DNA Genotek Inc.), respectively. DNA was quantified using Nanodrop ND-1000 spectrophotometer (NanoDrop Technologies) and checked on a 0.8% agarose gel, stained with ethidium bromide.

### Subjects

#### HHL family

**Fig. 1:**
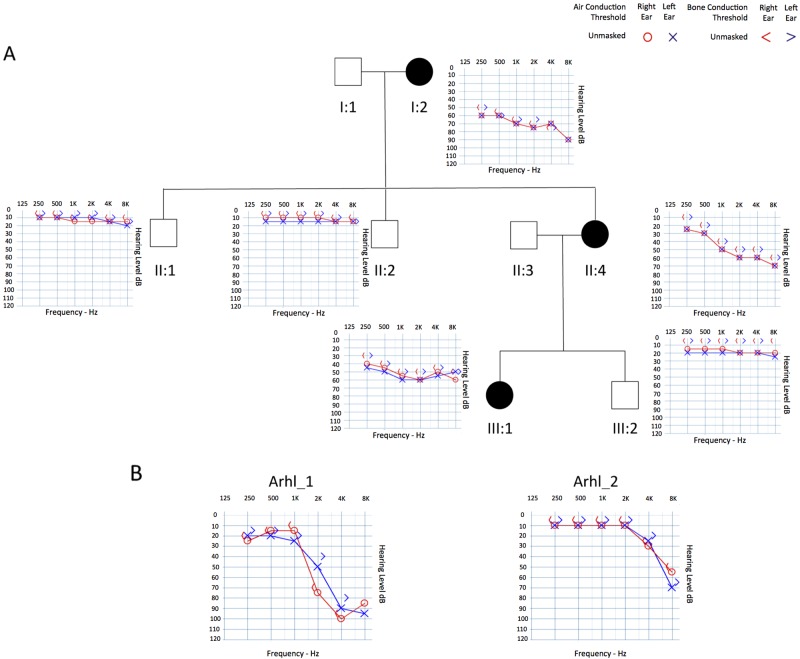
Pedigree and clinical features of the family and of the two ARHL patients. **a** Pedigree and audiograms of the Italian family carrying a stop variant in *SPATC1L*. Filled symbols represent affected individuals. **b** Audiograms of patients Arhl_1 and Arhl_2: the downward slope indicates that high frequencies are severely affected

An Italian family affected by early-onset HHL was enrolled in this study (Fig. 1a). Pure-tone audiometry and careful clinical evaluations of all patients were conducted. A detailed phenotypic description is reported in Supplementary S1.

#### ARHL patients

A cohort of 464 ARHL patients, derived from inbred (Friuli Venezia Giulia-FVG- Cohort, Carlantino Cohort) and outbred (Milan, Naples, Trieste) Italian populations, was analysed by targeted re-sequencing (TRS). This cohort consists of 258 males and 206 females, all of whom were over 50 years old and who had high-frequency bilateral HL that developed around the 5th decade of life. No defects of the external or middle ear, nor any vestibular problem or syndromic feature, were present.

#### Population-based cohort

DNA samples and phenotypic data of 604 healthy people from several rural communities located along the Silk Road countries were collected [9] (Figure S1). Audiometric tests and a clinical examination were carried out for each individual, as described in Supplementary S1.

#### Matched control samples

Whole genome sequencing data of 1071 healthy individuals (43% males, 57% females) from different Italian genetic isolates (INGI-Italian Network of Genetic Isolates) were used as an internal database. Among them, 113 subjects were used as controls for ARHL (individuals aged >50 y.o. with pure tone average (PTA) at the high frequencies <25 dB).

### Genetic analysis

#### HHL family

To reveal the genetic cause of HHL in the Italian family enrolled in this study (negative for variants in *GJB2*, *GJB6* and *MTRNR1*), subjects were first screened for 96 deafness-genes using our TRS (Targeted Resequencing) protocol [10]. Subjects I:2, II:1, II:2, II:3, II:4 and III:1 were then selected for whole exome sequencing (WES), carried out using the Ion Proton^TM^ platform (Life-Technologies). Briefly, 1 μg of genomic DNA was used to construct DNA libraries using the Ion AmpliSeq^TM^ Exome Kit. Libraries were sequenced with the Ion Proton^TM^ System (Life-Technologies), according to the manufacturer’s protocols. Read mapping and variant calling were performed using Ion TorrentSuite v4.0 software (Life-Technologies). Single nucleotide variants (SNVs) and small insertion and deletions (INDELs) were assessed using Variant Call Format (VCF) version 4.1 [11]. SNVs and INDELS were annotated with ANNOVAR [12] and filtered using exclusion criteria described in Figure S2. We then manually investigated the raw sequence reads for all the candidate variants using the Integrative Genomics Viewer (IGV) [13] to exclude false positive calls due to read misalignment. Finally, SNVs/INDELs that passed the filtering process were analysed by Sanger sequencing on a 3500 Dx Genetic Analyzer (Life-Technologies), using ABI PRISM 3.1 Big Dye terminator chemistry (Life-Technologies).

#### ARHL patients

A total number of 46 ARHL-candidate genes, including *SPATC1L* (NC_000021.8), were sequenced using Ion Torrent PGM™ (Life-Technologies). Genes were selected according to data from: (a) GWAS meta-analyses on isolated and outbred populations of European and Asian ancestry [4–6, 14], (b) literature updates and (c) animal models (unpublished data). The full list of genes with relevant references is available in Table S1. A total number of 464 ARHL patients have been analysed by TRS. Briefly, 10 ng of genomic DNA was used to construct DNA libraries using Ion AmpliSeq Library Kit 2.0 (Life-Technologies). Template ion sphere particles were prepared using Ion PGM Template OT2 200 kit and a single end 200 base-read sequencing run was carried out using Ion PGM sequencing 200 kit v2 (Life-Technologies), on Ion Torrent PGM (Life-Technologies). Ten indexed patients’ libraries were sequenced simultaneously on each Ion 318 Chip. Sequencing data were then analysed according to the Ion Torrent Suite^TM^ v3.6; SNVs and INDELs were collected into a standardized VCF version 4.1 and filtered according to criteria described in Figure S2. Variants were classified as ultra-rare (minor allele frequency (MAF < 0.001), rare (MAF < 0.01) or common (MAF > 0.01) based on frequencies reported in public databases. Finally, variants that most likely affect protein function (i.e., rare and ultra-rare variants predicted as damaging by all in silico predictor tools) were analysed by Sanger sequencing and tested in controls.

### Protein modelling and molecular dynamics simulations

The protein structure of human speriolin-like (SPC1L_HUMAN) was constructed using UniProt sequence (UniProt ID: Q9H0A9). Because no homologous structures are available for modelling, a new structure was built by applying iterative threading assembly refinement [15]. The quality of the wild-type model was assessed by methods previously reported [16]. In order to produce the mutants, Discovery studio (Accelrys Inc.) was used [17]. Wild-type and mutant proteins were used in the GROMACS simulation package to prepare molecular dynamics (MD) simulations by applying GROMOS96 force field [18–20]. Proteins were solubilized in the water model of SPC3 in a cubic box with a size of 1.5 nm [20]. The systems were neutralized by adding counter ions, and periodic boundary conditions were applied in all directions. The resulting systems contained from ~52,000 atoms to ~88,000 atoms. For long-range interactions, a twin range cut-off was used: 0.8 nm for van der Waals and 1.4 nm for electrostatic interactions. The LINCS [21] algorithm was used to constrain all bond lengths. To constrain the geometry of water molecules, the SETTLE [22] algorithm was applied. The systems were subjected to energy minimization using the steepest descent algorithm with the tolerance of 2000 Kj/mol/nm followed by 100 ps pre-equilibration and 10 ns of production MD simulations. This is with a time‐step of 2 fs at constant temperature (300 K), pressure (1 atm) and a number of particles, without any position restraints [23]. At every 100 ps, a structural snapshot was collected and the tools available within GROMACS were used for analysing the collected trajectories. The cluster analysis was utilized to select the representative structures from MD simulations. Each simulation produced 10,000 structures that were all grouped into clusters based on their structural deviation, in which the top-ranked cluster was chosen for representation, as it is a frequently occurring confirmation. The PyMOL package (www.pymol.org) was used for all the graphical representations. Sequence alignment of SPC1L_HUMAN with mouse speriolin (SPERI_MOUSE) (UniProt ID: Q148B6) was performed using ClustalO (http://www.clustal.org/).

### In vitro molecular cloning

The impact of the identified variants on mRNA and protein levels was tested by transient transfection in Hek293 cells using expression clones containing either the wild-type or the mutant cDNA. cDNAs were cloned into a pCMV6-Entry vector (Origene), which was Myc-tagged. Forty-eight hours after transfection total cell proteins and RNAs were prepared and analysed by Western blot and quantitative Real-Time PCR (qRT-PCR), respectively.

#### Western blot analysis and quantitative real-time PCR (qRT-PCR)

Hek293 cells were lysed in IPLS buffer (50 mM Tris-HCl pH7.5, 120 mM NaCl, 0.5 mM EDTA and 0.5% Nonidet P-40) supplemented with protease inhibitors (Roche). After sonication and pre-clearing, protein lysate concentration was determined by Bradford Assay (Biorad). An 8% polyacrylamide gel was used for protein electrophoresis. After blotting, membranes were blocked with 5% skim milk in Tris-buffered saline, 0.1% Tween 20 (TBST) and then incubated overnight with primary c-Myc Antibody 9E10 monoclonal (Santa Cruz) and HSP 90 monoclonal (Santa Cruz). Secondary antibodies (anti-mouse antibody (Santa Cruz)) were diluted in blocking buffer and incubated with the membranes for 45 min at room temperature. Proteins were detected with the ECL detection kit (GE Health Care Bio-Sciences). Housekeeping proteins were used as an internal control for protein loading as well as for reference in the western blotting analysis. RNA was extracted from cell pellets using a High Pure RNA Isolation Kit (Roche). Total RNA (1 μg) was reverse transcribed using Transcriptor First Strand cDNA Synthesis kit (Roche). qRT-PCR was performed using standard PCR conditions in a 7900HT fast real-time PCR System (Applied Biosystems) with Power SYBR Green PCR Master Mix (Thermo Fisher Scientific). Gene-specific primers were designed with Primer3Web software (http://bioinfo.ut.ee/primer3/) (Myc_For: 5′ AGCAGAAACTCATCTCAGAAG 3′; *SPATC1L*_human_cells_Rev: 5′ CGTGAACCTTCCGAAATCTG 3′). Expression levels were standardized to Neo gene expression and data were analyzed using the 2–ΔΔCT Livak Method [24]. All experiments were performed in biological triplicates.

### Candidate gene association study

DNA samples (*n* = 604) were genotyped with the Illumina Exome Chip. The analysis focused on functional SNPs belonging to the coding region of *SPATC1L*. Variant calling was refined using zCall [25], and quality control filters were applied taking into account standard parameters such as call rate > 0.99, Hardy-Weinberg equilibrium (*p* < 1e-08) and heterozygosity (FDR < 0.01). A candidate gene association study was performed for three different PTAs on common variants only (MAF > 0.01). Outliers, defined as subjects being more than three standard deviations from the mean of the phenotypic distribution, were excluded from the analysis. To account for population structure and relatedness, a linear mixed model with sex and age as covariates and the kinship matrix as the random effect was applied. The kinship matrix was computed over the whole set of available common SNPs (total ~80,000).

### *SPATC1L* tissue expression

Total RNA samples were extracted from wild-type CD1 mouse whole cochleae at post-natal day 3 (P3), P8, P12 and 2 month-old. Moreover, total RNA from different tissues including liver, spleen, lung, kidney, brain, testis and heart was extracted from 2-month old mice. The extraction was made using Direct-zol RNA MiniPrep Kit (Zymo Research). RNA was quantified using Nanodrop ND-1000 (NanoDrop Technologies). cDNAs were generated from 5 μg of total RNA using the Transcriptor First Strand cDNA Synthesis Kit (Roche) and were used for semi-quantitative RT-PCR (sqRT-PCR). Six microlitres of the RT products were used for PCR amplification. Gene-specific primers were designed with Primer3Web software (SPATC1L_1For: TACCTCAATGAGATCCAGAG; SPATC1L_1Rev:AACACATAGGCCAGGATACG). PCR reactions were optimized to 95 °C for 2 min, 30 amplification cycles at 95 °C for 10 s, 60 °C for 15 s, 72 °C for 4 s and a final extension of 1 min at 72 °C using Kapa HiFi HotStart ReadyMix PCR kit (Kapa Biosystem). Amplified products were checked on 2% agarose gels and visualized by ethidium bromide staining. β-Actin was used as internal control. Experiments were performed in biological triplicates.

## Results

### Familial HHL

In this study, an Italian family, displaying bilateral sensorineural symmetric progressive moderate-severe to profound hearing loss (Fig. 1a) was analysed by TRS and WES. TRS did not reveal any disease-causing variant (Supplementary S2). The overall mean-depth base coverage for WES was 108×, while on average 89% of the targeted region was covered at least 20-fold. A total of 91,597 variants were called among the six subjects included in the study. After variant filtering, we identified eight candidate SNVs. Three of them were excluded because of their frequency in our internal sequencing database (i.e., NGS data from over 1071 unrelated individuals). The remaining ones (Table S2) have been prioritized according to: (1) role of the gene based on literature research, (2) type of variant, and (3) segregation within the family. Four missense variants affecting genes already known to be associated with specific syndromes [26, 27] or with other traits not present in any of the affected members [28–30] were excluded from the analysis. The remaining nonsense variant, c.846C>G (NM_001142854.1), was present in *SPATC1L* (NC_000021.8) and leads to a premature stop codon with the loss of the last 58 amino acids of the protein (p.(Tyr282*)). This variant was not described in any public database and was not present in our internal database. Sanger sequencing demonstrated the correct segregation of this allele within the family (Figure S3A).

### Screening of SPATC1L in ARHL cases

TRS data of 464 ARHL patients were produced. A mean of 33.5 megabases of raw sequence data were available for each subject. The coverage, on 95% of the targeted region, was at least 20-fold, with a 270 fold mean-depth total coverage. On average 333 SNVs/INDELs were called for each patient. In particular, a frameshift duplication, c.340_343dupTTCA (p.(Lys115Ilefs*12)) (NM_001142854.1), was detected in Arhl_1 patient (70 y.o.), coming from Carlantino (an isolated village from Southern Italy) and showing moderate to profound HL (Fig. 1b). In this case, the availability of extended pedigrees allowed us to look for segregation of this allele in other family members. Despite the fact that we are dealing with a late onset disease where frequently there are no other living family members, we confirmed the absence of this variant in the only living, aged and healthy family member (i.e., first cousin aged 68 years old). A second allele, a missense variant c.656A>C (p.(Tyr219Ser)) (NM_001142854.1) was detected in patient Arhl_2 (55 y.o.), coming from Milan (Italy) and showing moderate to severe HL (Fig. 1b). Unfortunately, no living relatives were available for segregation analysis. All in silico predictor tools classified this allele as damaging. Both alleles were confirmed by Sanger sequencing (Figure S3B) and were the only ultra rare variants likely affecting protein function present in Arhl_1 and Arhl_2 cases. In particular, the c.340_343dupTTCA allele was described only in the gnomAD browser, with a MAF of 0.00001923, while the c.656A>C variant has never been reported. Moreover, both variants were not present in our internal database of NGS data.

All *SPATC1L* variants are available in the European Genome-Phenome Archive (EGA) database (https://ega-archive.org/studies/EGAS00001003047).

### Molecular modelling of protein

**Fig. 2:**
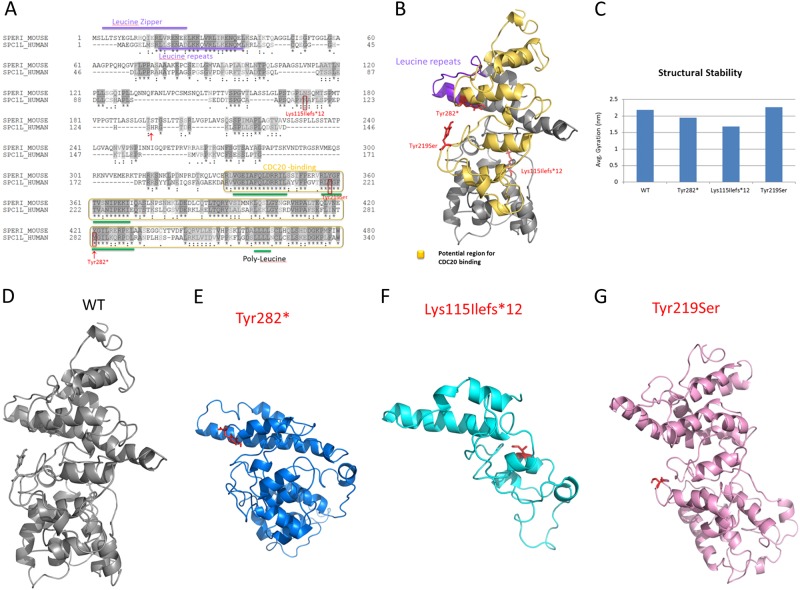
Sequence analysis and molecular modelling of the proteins. **a** Sequence alignment of human Speriolin-like protein (SPC1L_HUMAN, UniProt ID: Q9H0A9) with mouse Speriolin (SPERI_MOUSE, UniProt ID:Q148B6). **b** Molecular model of the human speriolin-like protein with potential functional regions. **c** Average radius of gyration of the four systems. **d–g** Representative structures of the wild type, c.846C>G (p.(Tyr282*)), c.340_343dupTTCA (p.(Lys115Ilefs*12)) and c.656A>C (p.(Tyr219Ser)) respectively, from molecular dynamics simulations. Here, the locations of the variants are represented as red sticks

To identify functional domains and to assess further the possible role of the speriolin-like protein, its sequence was compared to that of its paralog of known function, speriolin. In particular SPC1L_HUMAN and SPERI_MOUSE were compared (Fig. 2a). Sequence alignment shows a 70% sequence similarity with 129 identical residues. In SPERI_MOUSE the most conserved regions correspond to the N- and C-terminal regions that contain important functional domains, i.e., the leucine zipper and the binding region for cell division cycle protein 20 (Cdc20), respectively. According to sequence similarity, these domains are most likely present also in the human speriolin-like protein. Interestingly, all variants identified in our patients affect the C-terminus; in particular, c.656A>C (p.(Tyr219Ser)) occurs in a highly conserved region (RLYGFTV) while c.846C>G (p.(Tyr282*)) and c.340_343dupTTCA (p.(Lys115Ilefs*12)) cause the partial and total loss of this region, respectively. The impact of all variants was analysed by MD simulations with the representative structures. As expected, the wild-type showed a stable behaviour by maintaining secondary structures (Fig. 2d–g), while all mutants displayed abnormal behaviours. Overall MD of the SPC1L_HUMAN systems demonstrated: (a) reduced structural stability for the c.846C>G (p.(Tyr282*)) mutant in addition to the loss of part of the C-terminus (283–340) and mild alteration of the secondary structures at the N-terminus; (b) reduced structural stability for the c.340_343dupTTCA (p.(Lys115Ilefs*12)) mutant plus loss of ~75% of the protein leading to an abnormal protein isoform; (c) increased structural stability and rigidity for the c.656A>C (p.(Tyr219Ser)) mutant and slight conformational changes nearby the mutational spot.

### In vitro molecular cloning

**Fig. 3:**
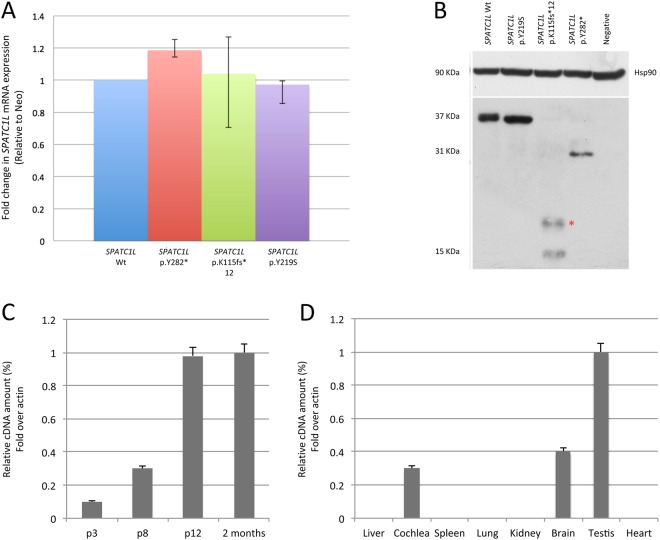
*SPATC1L* mRNA and protein levels in Hek293 transfected cells and *Spatc1l* expression in mouse whole cochlea and other tissues at different time points. **a** qRT-PCR analysis of relative mRNA expression of *SPATC1L* wild type and mutants after 48 h of transfection in Hek293 cells. Results are expressed as a fold-change of expression levels, and are normalized to the relative amount of the internal standard Neo. Error bars indicate 95% confidence intervals. **b** Western blot analysis of SPATC1L wild type and mutant proteins. Hsp90 was applied to determine equal loading. **c** The graph shows expression of *Spatc1l* in mouse whole cochlea at P3, P8, P12 and 2 months. Results are reported as fold change in gene expression over β actin, used as an internal control. The gene shows an age-related expression. **d** The graph shows *Spatc1l* expression at 2 months of age in different mouse tissues, including liver, cochlea, spleen, lung, kidney, brain, testis and heart. Results are reported as fold change in gene expression over β actin, used as an internal control

To investigate further the effect of *SPATC1L* variants on mRNA and protein levels, expression vectors containing either the wild-type *SPATC1L* cDNA or the mutant ones were used. qRT-PCR didn’t reveal any significant difference in the expression levels of all mutants compared to wild type (Fig. 3a). Western blot analysis confirmed the presence of all mutated protein isoforms (Fig. 3b). In particular, in the case of the missense variant of the Arhl_2 patient, c.656A>C (p.(Tyr219Ser)), a full-length protein was detected. On the contrary, in the case of the two truncating variants carried by the Arhl_1 patient and by the Italian family (c.340_343dupTTCA (p.(Lys115Ilefs*12)) and c.846C>G (p.(Tyr282*)) respectively), shorter protein isoforms were observed.

### Genetic association and expression studies

To investigate further the role of *SPATC1L* in hearing, a candidate gene association analysis in a healthy and completely independent cohort coming from the Caucasus and Central Asia (Silk Road) (https://www.ebi.ac.uk/ena/data/view/PRJEB7469) was performed. After filtering for quality parameters and MAF, only 3 SNPs (rs14378, rs113710653 and rs113146399) located in the *SPATC1L* coding region were available for the analysis. Results, including size estimates and *p*-values for each SNP and trait, are shown in Table 1. Statistically significant associations with 3 *SPACT1L* SNPs, were obtained for pure tone average of low frequencies (PTAL) (i.e., 0.25, 0.5 and 1 kHz), (rs14378, *p* = 0.031556621; rs113710653, *p* = 0.005643919; rs113146399, *p* = 0.026677654) and PTA of middle frequencies (PTAM) (i.e., 0.5, 1 and 2 kHz) (rs14378, *p* = 0.04347863; rs113710653, *p* = 0.00802626; rs113146399, *p* = 0.03660835). A boxplot of PTAL residuals for the three genotypes of the most associated SNP (rs113710653, *p* = 0.00564) is given in Figure S4. Individuals carrying the alternative allele have worse hearing, taking into account sex and age differences. In particular, homozygous TT subjects show at PTAL and PTAM an increased dB threshold (+2.3 dB and 3.27 on average respectively) compared to the wild type (CC) ones (OR = 1.17, 95% CI = [1.05–1.31] for PTAL and OR = 1.20, 95% CI = [1.05–1.38] for PTAM). None of these SNPs match with any known eQTL region, according to the GTex Database (http://www.gtexportal.org/home/). To support further the genetic results, gene expression studies on CD1 mice were performed. These studies revealed the expression of *Spatc1l* in mouse whole cochlea increasing with age from P3 to P12 (Fig. 3c), which corresponds to the development of auditory perception. Public databases, i.e., SHIELD (https://shield.hms.harvard.edu/index.html), gEAR (http://gear.igs.umaryland.edu/) and Mouse Genome Informatics (MGI) (http://www.informatics.jax.org/), confirmed gene expression in mouse inner ear, even though at low levels. In particular, according to SHIELD, *Spatc1l* is more abundantly expressed in hair cells compared to non-hair cells during mouse inner ear development, and in adult mice it is more expressed in cochlear outer hair cells compared to inner hair cells. Finally it is also detected in the vestibular and spiral ganglion at different developmental stages. In agreement with literature data, *Spatc1l* expression was also detected in brain and testis from 2 month-old mice (Fig. 3d).

**Table 1:** Results of the candidate gene association analysis

Trait	SNP	Chr	Position	Effect allele	Other allele	Eff. all. freq	*N*	Beta	SE beta	*P*-value
PTAL	rs14378	21	47581423	T	C	0.054	598	0.05334218	0.02481052	0.031556621
PTAL	rs113710653	21	47581835	T	C	0.039	590	0.07971061	0.02879945	0.005643919
PTAL	rs113146399	21	47581949	T	C	0.044	598	0.06031144	0.02721387	0.026677654
PTAM	rs14378	21	47581423	T	C	0.054	597	0.06057786	0.03000266	0.04347863
PTAM	rs113710653	21	47581835	T	C	0.040	589	0.09218281	0.03477333	0.00802626
PTAM	rs113146399	21	47581949	T	C	0.044	597	0.06870397	0.03287106	0.03660835
PTAH	rs14378	21	47581423	T	C	0.054	604	0.006119463	0.04790555	0.8983546
PTAH	rs113710653	21	47581835	T	C	0.039	596	0.02570644	0.05588673	0.6455349
PTAH	rs113146399	21	47581949	T	C	0.044	604	−0.004327187	0.05277132	0.9346476

*PTAL* pure tone average of low frequencies, *PTAM* pure tone average of medium frequencies, *PTAH* pure tone average of high frequencies, *Eff. all. freq* effect allele frequency, *Beta* effect size estimate, *SE Beta* effect size standard error

## Discussion

Here we propose, for the first time, the involvement of *SPATC1L* in hearing function and loss. *SPATC1L* encodes two different isoforms of the speriolin-like protein, whose function is still unknown. A study from Lecat et al. [31], revealed that SPC1L_HUMAN localises in the cytoplasm, in the nucleus and in the perinuclear region in Hek293 cells, and after activation of the NK2 receptor it moves to cellular junctions, suggesting a possible role of this protein in cellular junction formation. One major type of cell junctions are gap junctions, existing extensively in different parts of the cochlea and having many functions herein [32]. To date, apart from the role of connexins, little is known about functions of other proteins in cellular junctions. Considering the relevance of these cellular components in hearing function, the role of *SPATC1L* and other related proteins warrants further investigation. It has also been shown that *SPATC1L* modulates the response of human cells to alkylating agents, having a protective effect. Thus, decreased gene expression correlates with an increased sensitivity to alkylating agents, such as N-methyl-N’-nitro-N-nitrosoguanidine (MNNG) [33]. Further investigations on how *SPATC1L* plays this role will help in understanding if it is involved in processes that guarantee cell survival in response to damaging agents or oxidative stress, a presumed major cause of ARHL and hearing dysfunction. Accordingly, some ARHL candidate genes are involved in oxidative stress response and mitochondrial dysfunction [34]. In order to test the effect of all *SPATC1L* variants, MD simulations were performed. Overall MD suggested that all mutated alleles affect protein stability and secondary structural elements, which are essential for protein function [35-39]. In particular, we observed severe structural changes leading to incomplete isoforms, with partial or entire loss of the C-terminus (i.e., c.340_343dupTTCA (p.(Lys115Ilefs*12)) and c.846C>G (p.(Tyr282*)) mutants), while for the c.656A>C (p.(Tyr219Ser)) mutant a disturbance of a highly conserved region of the C-terminus and an increased structural rigidity were observed. Considering that the C-terminus of SPC1L_HUMAN contains important functional domains, the loss/alteration of this region might be highly deleterious. The impact of all variants was also evaluated by in vitro molecular cloning. In particular, Western blot analysis revealed the presence of all mutated protein isoforms that, according to our protein modelling results, are likely to be dysfunctional, thus leading to altered protein function. Interestingly, in the case of the frameshift duplication (patient Arhl_1), two different bands were detected: a higher one, with the expected molecular weight, and a smaller one. Considering the huge impact of this variant on protein structure, it is highly probable that the truncated isoform will be rapidly degraded, thus the shorter band detected at Western blot analysis may represent a pattern of protein degradation. Moreover, results from expression studies reveal that *SPATC1L* expression increases during developmental stages in hearing and then it remains stable until adult age, suggesting its possible involvement in the development and maintenance of the auditory system. A remarkable expression was also detected in both brain and testis indicating a possible role also in these tissues despite the absence of any clinical symptoms related to these two organs in the cases reported herein. Considering all these findings, our hypothesis suggests that these variants can lead to slightly different phenotypes. In fact, the severe to profound phenotype in the HHL patients is due to a nonsense allele causing a reduced stability and integrity of the protein and affecting both isoforms of the gene, thus leading to more deleterious consequences. Accordingly, a similar severe phenotype was present in the ARHL patient carrying another disruptive allele (i.e., the frameshift duplication) despite affecting the coding region of only one of the two isoforms. As expected, a milder phenotype is due to the presence of the missense variant in the second ARHL patient. In conclusion, the amount of genetic data presented herein, together with functional and expression data, plus an interesting genotype–phenotype correlation, suggest a role of *SPATC1L* in the auditory system.

## Electronic supplementary material

Supplementary S1

Supplementary S2

Table S1

Table S2

Figure S1

Figure S2

Figure S3

Figure S4

Additional Supplementary file
