# Supplementary material for: Next-generation sequencing identified SPATC1L as a possible candidate gene for both early-onset and age-related hearing loss
Source: Eur J Hum Genet. 2018 Sep 3;27(1):70–9. doi: 10.1038/s41431-018-0229-9 (PMC6303261; doi:10.1038/s41431-018-0229-9)
Supplement: Supplementary file 1 — Supplementary S1 [file 41431_2018_229_MOESM1_ESM.docx]

**Supplementary S1. Clinical information and inclusion criteria of the recruited patients.**

**1) Phenotypic evaluation of the Italian HHL family.**

All the affected family members underwent a careful clinical examination.

Pure-tone audiometry evaluation showed bilateral sensorineural symmetric progressive hearing loss. Conductive hearing impairment due to external and middle ear defects was excluded thanks to bone conduction thresholds (similar to the air conduction thresholds), tympanometry (all affected patients present with type A tympanogram) and acoustic reflex (ipsilateral and contralateral in both ears ranging from 95 to 105 dB at 500Hz, 1KHz, 2KHz, 4KHz). Clinical examination and clinical history were negative for the presence of vestibular dysfunction as well as for any kind of syndromic hearing loss. The presence of Cytomegalovirus infections and autoimmune phenotypes was also ruled out.

The hearing thresholds and the age of onset show a worsening phenotype through the 3 generations. Figure 1A displays the latest audiological examination performed when subjects were 59 (I:2), 27 (II:4) and 2 (III:1) years old, respectively. Briefly, the grandmother (I:2) displays a moderate-severe to profound hearing impairment developed in the third decade of life and progressing over the years, leading to the need of hearing aids at the age of 50. The proband’s mother (II:4) shows a down-sloping audiometric configuration (normal to moderate hearing loss at the low frequencies and moderately severe at the medium-high frequencies) with an onset in the first decade of life and an important progression during the past years. Finally, the proband (III:1) shows a moderately severe hearing loss worsening at medium-high frequencies. The proband’s brother (III:2), and two uncles (II:1, II:2) show normal hearing.

**2) Phenotypic evaluation and inclusion criteria of the population-based cohort.**

Each of the 604 healthy individuals underwent an accurate audiometric test. From the six hearing thresholds values collected, three pure tone averages of air-conduction thresholds (PTA) were calculated: PTAL at low frequencies (0.25, 0.5 and 1 kHz), PTAM at middle frequencies (0.5, 1 and 2 kHz) and PTAH at high frequencies (4 and 8 kHz). These three pure tone averages were used as quantitative traits in the association analysis. Moreover, to avoid non-genetic variations in the hearing phenotype (e.g. monolateral hearing loss), the best hearing ear was considered for the analyses. Subjects affected by syndromic forms or other systemic illnesses linked with sensorineural hearing loss were excluded.
