# Supplementary material for: Next-generation sequencing identified SPATC1L as a possible candidate gene for both early-onset and age-related hearing loss
Source: Eur J Hum Genet. 2018 Sep 3;27(1):70–9. doi: 10.1038/s41431-018-0229-9 (PMC6303261; doi:10.1038/s41431-018-0229-9)
Supplement: Supplementary file 2 — Supplementary S2 [file 41431_2018_229_MOESM2_ESM.docx]

**Supplementary S2. TRS data of the Italian HHL family.**

TRS data of six subjects (I:2, II:1, II:2, II:3, II:4 and III:1) were produced. A mean of 216,8 megabases of raw sequence data were available for each subject. The coverage, on 97% of the targeted region, was at least 20-fold, with a 389,6 fold mean-depth total coverage. On average 517 SNVs/INDELs were called for each patient.

After applying the same filtering procedure described for WES data, it was not possible to identify any variant segregating within the family.
