# Supplementary material for: Next-generation sequencing identified SPATC1L as a possible candidate gene for both early-onset and age-related hearing loss
Source: Eur J Hum Genet. 2018 Sep 3;27(1):70–9. doi: 10.1038/s41431-018-0229-9 (PMC6303261; doi:10.1038/s41431-018-0229-9)
Supplement: Supplementary file 3 — Table S1 [file 41431_2018_229_MOESM3_ESM.docx]

**Table S1.** **List of genes included in our TRS panel (hg19 assembly)**

| **Gene** | **Chr** | **Start** | **End** | **Info** | **Reference** |
| --- | --- | --- | --- | --- | --- |
| *ANK2* | 4 | 113739265 | 114304896 | GWAS | Girotto et al 2011; 2014 |
| *ARSG* | 17 | 66255323 | 66418872 | GWAS | Girotto et al 2011; 2014 |
| *ATOH1* | 4 | 94750042 | 94751221 | Literature | Stojanova et al 2015 |
| *C2orf43* | 2 | 20883788 | 21022882 | Literature | Currall et al. abstract book ARO meeting 2014 |
| *CDH13* | 16 | 82660408 | 83830204 | GWAS | Girotto et al 2011; 2014 |
| *CEACAM16* | 19 | 45202421 | 45213986 | Literature | Cheatham et al. 2014 |
| *CEP104* | 1 | 3728645 | 3773778 | GWAS | Girotto et al 2011 |
| *CLRN1* | 3 | 150643950 | 150690786 | Literature | Philips et al. 2013 |
| *CSMD1* | 8 | 2792875 | 4852494 | GWAS | Girotto et al 2011; 2014 |
| *DCLK1* | 13 | 36345478 | 36705443 | GWAS | Girotto et al 2011; 2014 |
| *ELMOD1* | 11 | 107461817 | 107537505 | Literature | Johnson et al 2012 |
| *EPS8* | 12 | 15773092 | 16035263 | Literature | Behlouli et al 2014 |
| *EVI5* | 1 | 92974253 | 93257961 | GWAS | Girotto et al 2011; 2014 |
| *FABP3* | 1 | 31838472 | 31849697 | Literature | Sainto-Saito et al. 2010 |
| *FN1* | 2 | 216225163 | 216300895 | Literature | Song et al 2013 |
| *FOXO3* | 6 | 108881038 | 109005977 | Literature | Gilels et al 2013 |
| *GABRG3* | 15 | 27216429 | 27778373 | GWAS | Girotto et al 2011; 2014 |
| *GRM8* | 7 | 126078652 | 126893348 | GWAS | Girotto et al 2011; 2014 |
| *HMGA2* | 12 | 66217911 | 66360075 | Literature | Smeti et al 2014 |
| *IGF1* | 12 | 102789645 | 102874423 | Literature | de Iriarte Rodríguez et al 2015 |
| *INSM1* | 20 | 20348765 | 20351590 | Literature | Lorenzen et al 2015 |
| *ITFG2* | 12 | 2921788 | 2968957 | Internal analysis | personal communication |
| *KCNQ1* | 11 | 2465914 | 2870339 | Literature | Chang et al 2015 |
| *PCDH20* | 13 | 61983818 | 62002220 | GWAS | Vuckovic et al. 2015 |
| *PTPRCAP* | 11 | 67202981 | 67205538 | Internal analysis | personal communication |
| *PTPRD* | 9 | 8314246 | 10612723 | GWAS | Girotto et al 2011; 2014 |
| *RIMBP2* | 12 | 130880682 | 131200826 | GWAS | Girotto et al 2011; 2014 |
| *RYR3* | 15 | 33603163 | 34158303 | Literature | Liang et al 2009 |
| *SEPT7* | 7 | 35840542 | 35944917 | Literature | Yoshida et al 2012 |
| *SIK3* | 11 | 116714118 | 116969153 | GWAS | Wolber et al |
| *SLC16A6* | 17 | 66263167 | 66287408 | GWAS | Girotto et al 2011; 2014 |
| *SLC25A21* | 14 | 37147636 | 37642071 | Literature | Maguire et al 2014 |
| *SLC28A3* | 9 | 86890372 | 86955672 | GWAS | Vuckovic et al 2015 |
| *SLC44A2* | 19 | 10713133 | 10755235 | Literature | Beyer et al 2011 |
| *SLC9A3R1* | 17 | 72744791 | 72765492 | Literaure | Shin J-B et al. 2013 |
| *SMARCA4* | 19 | 11071598 | 11176071 | Internal analysis | personal communication |
| *STRN* | 2 | 37070783 | 37193615 | Internal analysis | personal communication |
| *TRPA1* | 8 | 72932152 | 72987852 | Literature | Stepanyan et al 2011 |
| *TRPM4* | 19 | 49660998 | 49715093 | Literature | Sakuraba et al 2014 |
| *WARS2* | 1 | 119573839 | 119683294 | ENU Mice | unpublished |
| *WBP2* | 17 | 73841780 | 73852588 | Literature | Buniello et al 2016 |
| *WDR1* | 4 | 10075963 | 10118573 | Literature | Song et al 2013 |
| *XIRP2* | 2 | 167744997 | 168116263 | Literature | Scheffer et al. 2015 |
| *SPATC1L* | 21 | 47581062 | 47605167 | Hearing loss family | present study |
| *TSP1* | 15 | 39873280 | 39889668 | Literature | Mendus et al 2014 |
| *TSP2* | 6 | 169615875 | 169654137 | Literature | Mendus et al 2014 |
|  |  |  |  |  |  |
|  | | | | |  |
|  |  |  |  |  |  |
