# Supplementary material for: Next-generation sequencing identified SPATC1L as a possible candidate gene for both early-onset and age-related hearing loss
Source: Eur J Hum Genet. 2018 Sep 3;27(1):70–9. doi: 10.1038/s41431-018-0229-9 (PMC6303261; doi:10.1038/s41431-018-0229-9)
Supplement: Supplementary file 4 — Table S2 [file 41431_2018_229_MOESM4_ESM.docx]

**Table S2. List of mutations found with whole exome sequencing in the Italian family.**

| **hg19 position** | chr1:103540302 | chr12:14792840 | chr18:74649238 | chr21:47581470 | chr4:162459343 |
| --- | --- | --- | --- | --- | --- |
| **Reference allele** | T | G | C | G | A |
| **Alternative allele** | G | A | G | C | C |
| **Gene** | *COL11A1* | *GUCY2C* | *ZNF236* | *SPATC1L* | *FSTL5* |
| **MIM** | 604841 | 614665; 614616 | NA | NA | NA |
| **GenBank** | NM_001190709 | NM_004963 | NM_007345 | NM_001142854 | NM_001128427 |
| **cDNA change** | c.523A>C | c.2113C>T | c.4715C>G | c.846C>G | c.1284T>G |
| **Amino acid change** | p.(Thr175Pro) | p.(Arg705Cys) | p.(Ala1572Gly) | p.(Tyr282*) | p.(Phe428Leu) |
| **dbSNP rs ID** | NA | rs771103086 | NA | NA | NA |
| **N° ExAC European (non-Finnish) allele** | 0 | 0 | 0 | 0 | 0 |
| **ExAC European (non-Finnish) MAF** | 0 | 0 | 0 | 0 | 0 |
| **ExAC all MAF** | 0 | 0 | 0 | 0 | 0 |
| **MAF in-house exomes** | 0 | 0 | 0 | 0 | 0 |
| **MAF in-house WGS** | 0 | 0 | 0 | 0 | 0 |
| **GERP score** | 5,73 | 4,69 | 5,81 | 1,47 | -4,5 |
| **phyloP100way_vertebrate** | 3,37 | 5,938 | 6,46 | -0,181 | 1,569 |
| **Fathmm** | Tolerated | Tolerated | Tolerated | NA | Tolerated |
| **MutationAssessor** | Medium | Medium | Low | NA | Neutral |
| **MutationTaster** | Disease causing | Disease causing | Disease causing | Disease causing | Disease causing |
| **Polyphen2 HVAR** | Possibly damaging | Probably damaging | Probably damaging | NA | Probably damaging |
| **SIFT** | Deleterious | Deleterious | Tolerated | NA | Tolerated |

Abbreviations are as follows: NA, not available; MIM: MIM number; MAF: minor allele frequency; WGS: whole genome sequencing.
