# Supplementary figures and images for: Next-generation sequencing identified SPATC1L as a possible candidate gene for both early-onset and age-related hearing loss
Source: Eur J Hum Genet. 2018 Sep 3;27(1):70–9. doi: 10.1038/s41431-018-0229-9 (PMC6303261; doi:10.1038/s41431-018-0229-9)

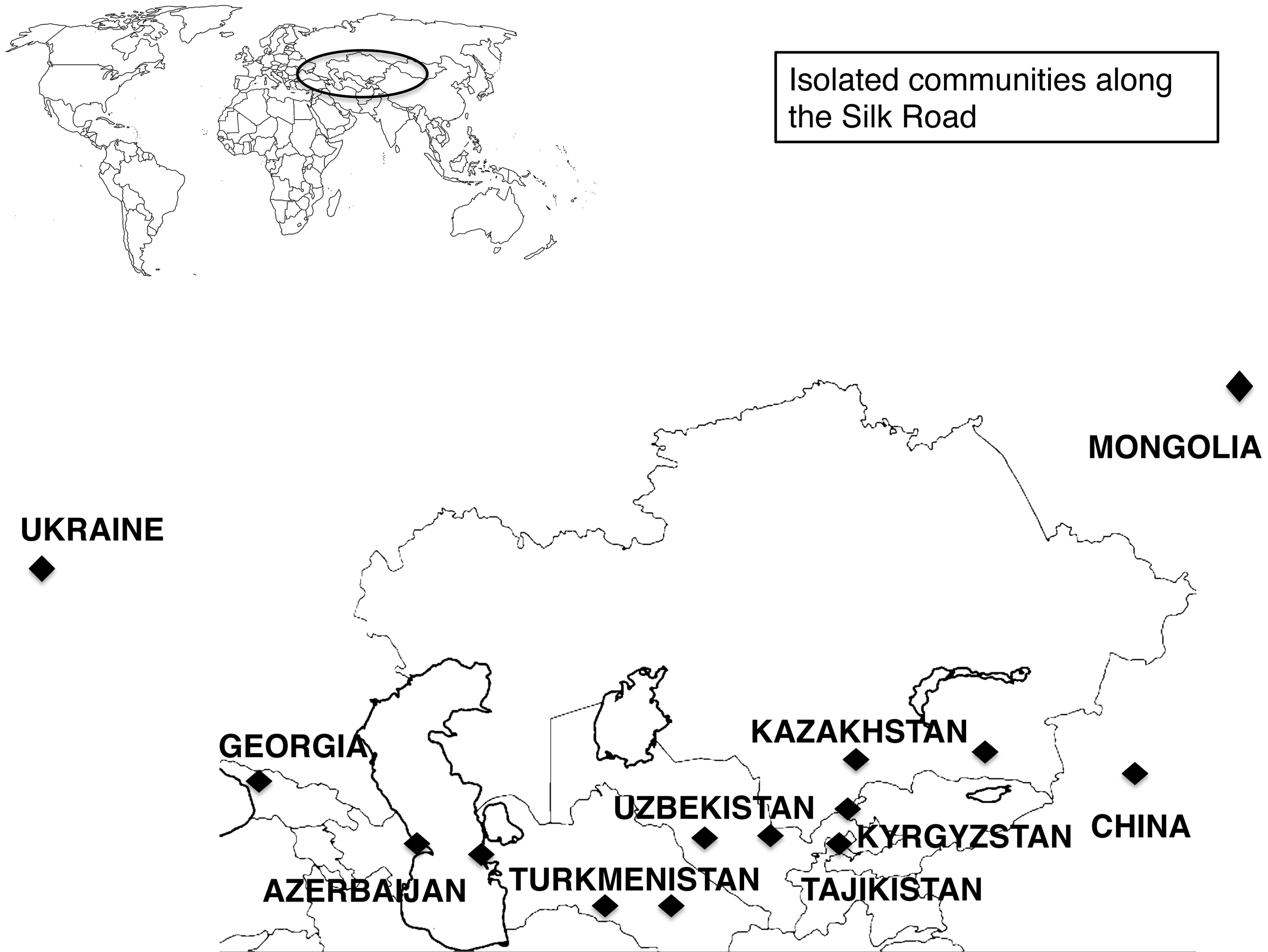

Supplement: Supplementary file 5 — Figure S1 [file 41431_2018_229_MOESM5_ESM.jpg]

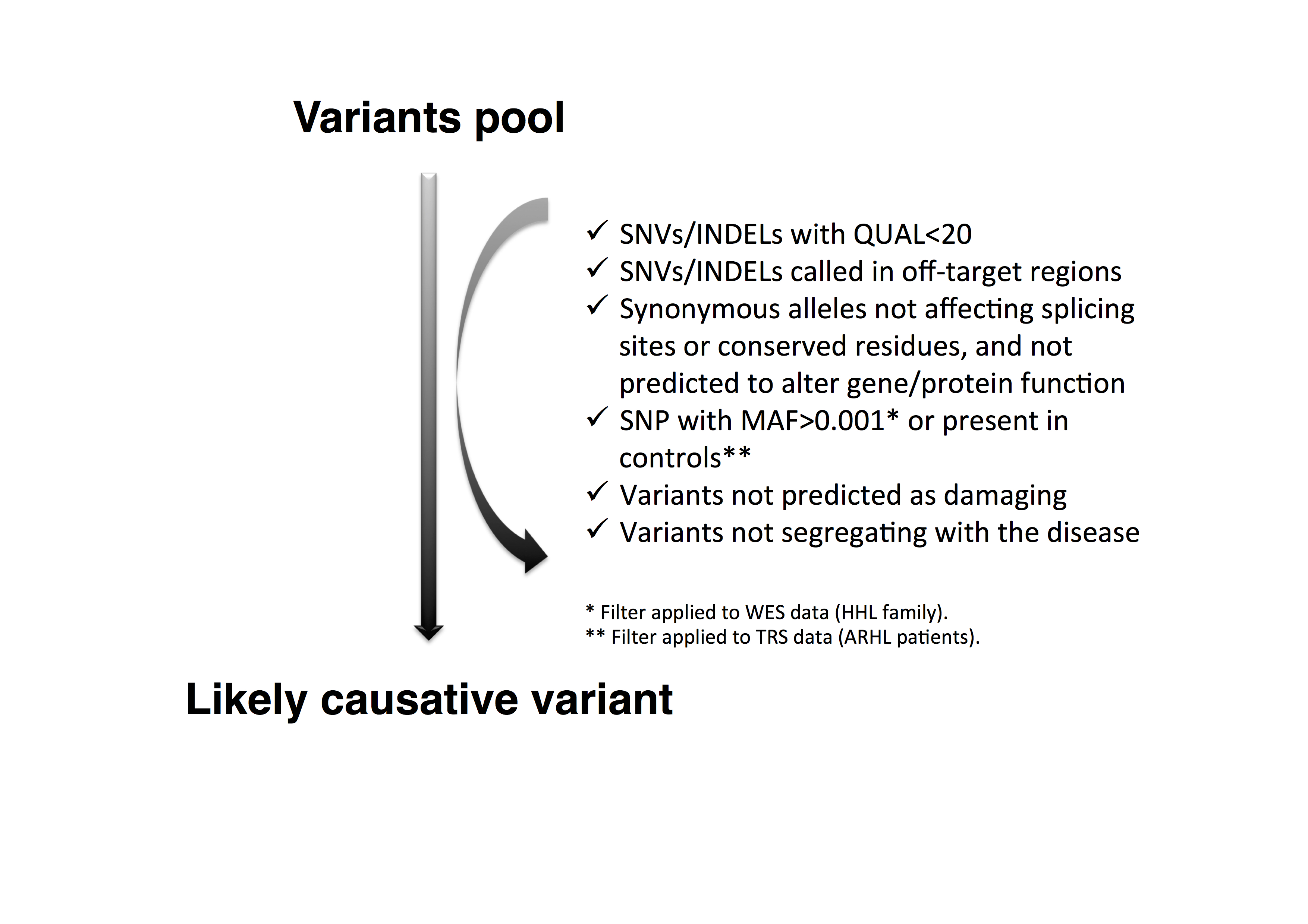

Supplement: Supplementary file 6 — Figure S2 [file 41431_2018_229_MOESM6_ESM.jpg]

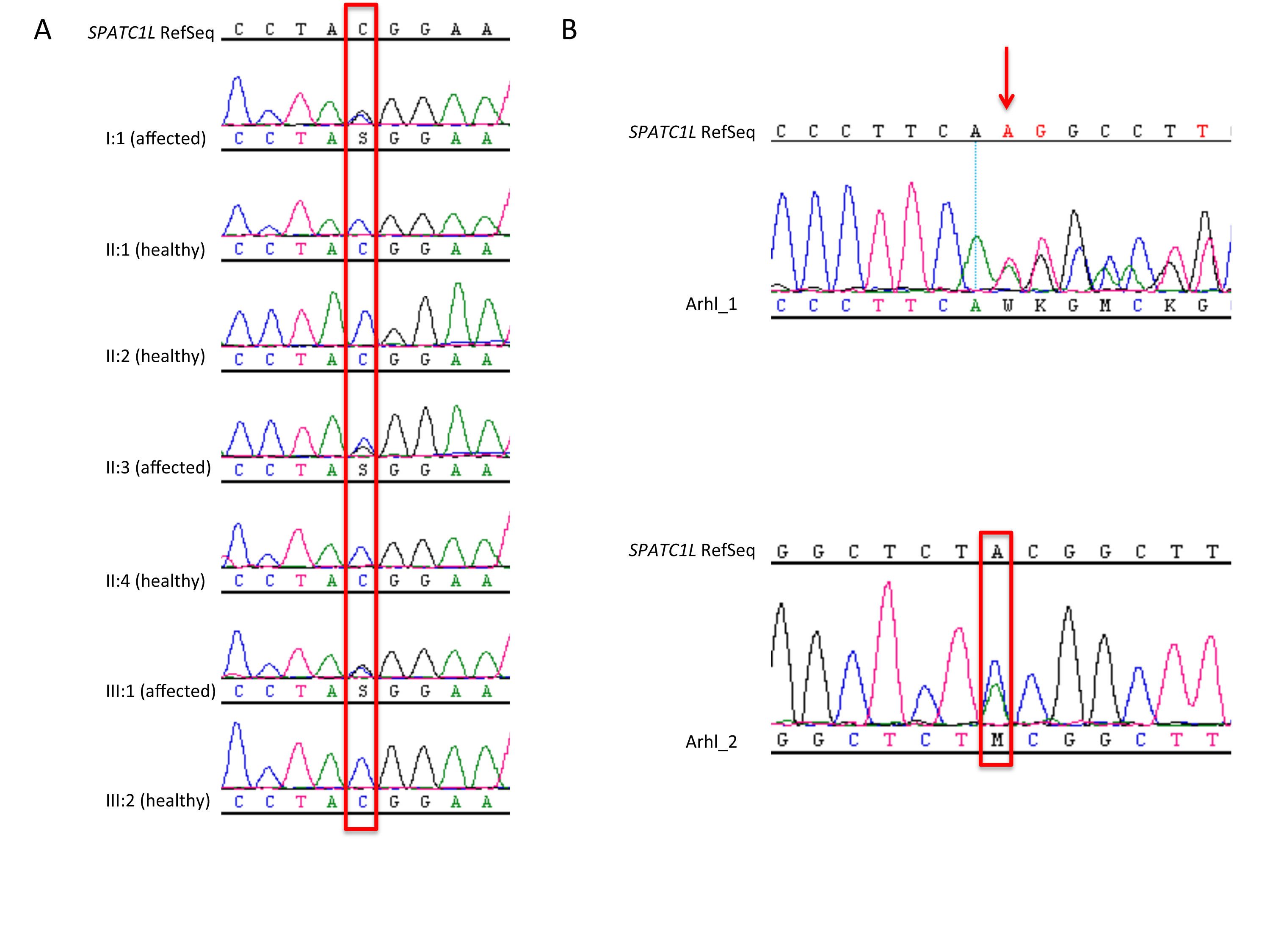

Supplement: Supplementary file 7 — Figure S3 [file 41431_2018_229_MOESM7_ESM.jpg]

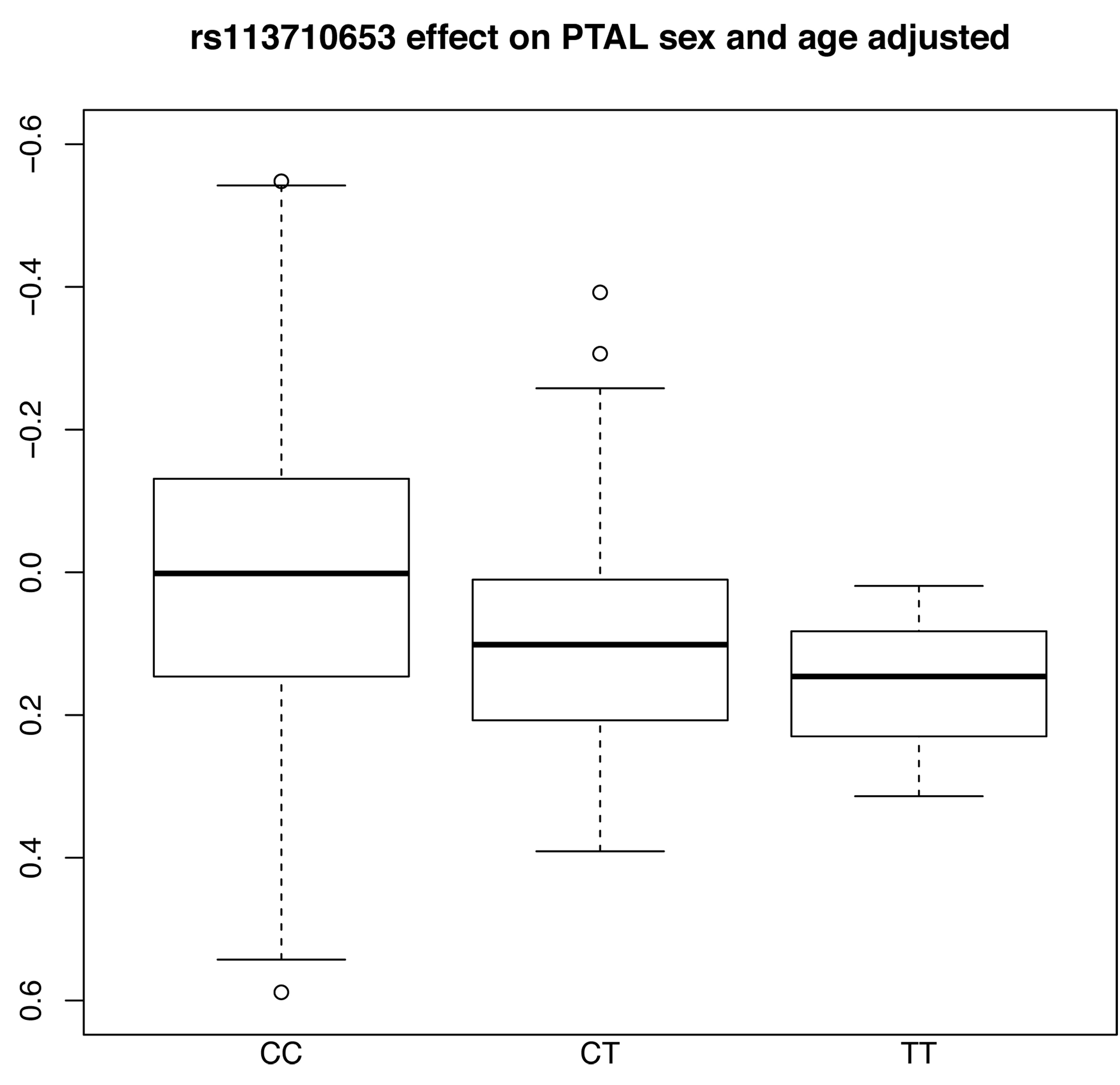

Supplement: Supplementary file 8 — Figure S4 [file 41431_2018_229_MOESM8_ESM.jpg]
