# Supplementary material for: Next-generation sequencing identified SPATC1L as a possible candidate gene for both early-onset and age-related hearing loss
Source: Eur J Hum Genet. 2018 Sep 3;27(1):70–9. doi: 10.1038/s41431-018-0229-9 (PMC6303261; doi:10.1038/s41431-018-0229-9)
Supplement: Supplementary file 9 — Additional Supplementary file [file 41431_2018_229_MOESM9_ESM.docx]

**Supporting Figures legends**

**Figure S1. Map of the Silk Road cohorts**

The figure shows the location of the communites used in this study for the statistical association analysis with *SPATC1L* exonic variants.

**Figure S2. Diagram of the filtering strategy applied to WES and TRS data.**

Next generation sequencing data have been filtered according to the following exclusion criteria: 1) SNVs/INDELs with QUAL<20; 2) SNVs/INDELs called in off-target regions; 3) SNVs leading to synonymous amino acids substitutions not predicted to affect gene/protein function by any disease predictor tools (SIFT, Polyphen2, MutationTaster, LRT) ^1–4^ and not affecting splicing sites or highly conserved residues (PhyloP) ^5^; 4) Variants with a minor allele frequency>0.001 (NCBI dbSNP build142 (<https://www.ncbi.nlm.nih.gov/projects/SNP/>), 1000 Genomes Project (http://www.1000genomes.org/), NHLBI Exome Sequencing Project (ESP) Exome Variant Server (<http://evs.gs.washington.edu/ESV/>), ExAC Browser (<http://exac.broadinstitute.org/about>) and gnomAD browser (<http://gnomad.broadinstitute.org/>)), or present in control samples; 5) Variants not predicted as damaging; 6) Variants not segregating within the family in accordance with a dominant pattern of inheritance (heterozygous variants shared by all the affected individuals and not present in any of the healthy subjects).

**Figure S3. DNA sequence chromatograms.**

The figure displays DNA sequence chromatograms showing the nucleotide variant identified in the Italian Family (a) and in patients Arhl_1 and Arhl_2 (b).

**Figure S4. rs113710653 effect on PTAL sex and age adjusted.**

The figure displays boxplots for the PTAL trait divided by genotype groups for rs113710653 and adjusted by sex and age in the SR cohort. As shown in the figure, individuals with the TT genotype have worse hearing function.

**References**

1 Ng PC, Henikoff S. SIFT: Predicting amino acid changes that affect protein function. *Nucleic Acids Res* 2003; **31**: 3812–4.

2 Adzhubei I, Jordan DM, Sunyaev SR. Predicting functional effect of human missense mutations using PolyPhen-2. *Curr Protoc Hum Genet* 2013; **Chapter 7**: Unit7.20.

3 Schwarz JM, Rödelsperger C, Schuelke M, Seelow D. MutationTaster evaluates disease-causing potential of sequence alterations. *Nat Methods* 2010; **7**: 575–6.

4 Chun S, Fay JC. Identification of deleterious mutations within three human genomes. *Genome Res* 2009; **19**: 1553–1561.

5 Pollard KS, Hubisz MJ, Rosenbloom KR, Siepel A. Detection of nonneutral substitution rates on mammalian phylogenies. *Genome Res* 2010; **20**: 110–21.
